# Integrated care through disease-oriented clinical care pathways: experience from Japan’s regional health planning initiatives

**Published:** 2011-09-23

**Authors:** Etsuji Okamoto, Masaki Miyamoto, Kazuhiro Hara, Jun Yoshida, Masaki Muto, Aizan Hirai, Haruyuki Tatsumi, Masaaki Mizuno, Hiroshi Nagata, Daisuke Yamakata, Hiroshi Tanaka

**Affiliations:** National Institute of Public Health, 2-3-6, Minami, Wako-shi, Saitama 351-0197, Japan; Hyogo Medical College, Department of Medical Informatics, 1-1 Mukogawa-cho, Nishinomiya-shi, Hyogo 663-8501, Japan; Kagawa University, Seto Inland Sea Regional Research Center, Saiwaicho 1-1, Takamatsu City, Kagawa 760-8521, Japan; Japan Labour Health and Welfare Organization CHUBU-ROSAI Hospital, 1-10-6 Komei, Minato-ku, Nagoya-shi, Aichi 455-8530, Japan; International Health and Welfare University Graduate School, 1-3-3 Minami-Aoyama, Minato-ku, Tokyo 107-0062, Japan; Chiba Prefectural Togane Hospital, 1229 Daiho, Togane-shi, Chiba 283-8588, Japan; Sapporo Medical School, Department of Anatomy, Minami-1-jo, Nishi-17-chome, Chuo-ku, Sapporo-shi, Hokkaido 060-8556, Japan; Nagoya University Hospital Center for Advanced Medicine and Clinical Research, 65 Tsurumai, Showa-ku, Nagoya-shi, Aichi 466-8560, Japan; Nagahama Bio University, Department of Computer Bioscience, 1266 Tamura-machi, Nagahama-shi, Shiga 526-0829, Japan; Kagawa University, Department of Medical Information, 1750-1 Ikedo, Miki-cho, Kida-gun, Kagawa 761-0793, Japan; Tokyo Medical and Dental University, Department of Bioinformatics, 1-5-45 Yushima, Bunkyo-ku, Tokyo 113-8519, Japan

**Keywords:** regional health planning, disease management, critical path, electronic health record, care pathways, Japan

## Abstract

**Introduction:**

In April 2008, Japan launched a radical reform in regional health planning that emphasized the development of disease-oriented clinical care pathways. These ‘inter-provider critical paths’ have sought to ensure effective integration of various providers ranging among primary care practitioners, acute care hospitals, rehabilitation hospitals, long-term care facilities and home care.

**Description of policy practice:**

All 47 prefectures in Japan developed their Regional Health Plans pursuant to the guideline requiring that these should include at least four diseases: diabetes, acute myocardial infarction, cerebrovascular accident and cancer. To illustrate the care pathways developed, this paper describes the guideline referring to strokes and provides examples of the new Regional Health Plans as well as examples of disease-oriented inter-provider clinical paths. In particular, the paper examines the development of information sharing through electronic health records (EHR) to enhance effective integration among providers is discussed.

**Discussion and conclusion:**

Japan’s reform in 2008 is unique in that the concept of ‘disease-oriented regional inter-provider critical paths’ was adopted as a national policy and all 47 prefectures developed their Regional Health Plans simultaneously. How much the new regional health planning policy has improved the quality and outcome of care remains to be seen and will be evaluated in 2013 after the five-year planned period of implementation has concluded. Whilst electronic health records appear to be a useful tool in supporting care integration they do not guarantee success in the application of an inter-provider critical path.

## Introduction

Japan’s health care system is financed by universal coverage of health insurance with strong price control by the government and is provided by hospitals and clinics in the predominantly private sector with little control over utilization. It is an outlier in terms of international comparison among OECD countries [1]. Japan has the highest number of hospital beds per population (8.2 per thousand population vs. OECD average 3.8) with the longest length of stay (LOS) (19.0 days vs. OECD average 6.5 days), the highest utilization of physician consultations (13.6 per capita annually vs. OECD average 6.8) and by far the highest number of MRI and CT scans (40.1 and 92.6 per million population vs. OECD average 11.0 and 22.8, respectively). However, Japan’s health care expenditure is kept relatively low in relation to its GDP (Gross Domestic Product) (Japan: 8.1% vs. OECD average 8.9%).

The discrepancy between the high utilization of health care resources and relatively low health care expenditure can be explained by the strong and universal price control by the government. Such price control is a strength of Japan’s national health insurance system in guaranteeing the equal and universal access to health care. However, Japan’s health care has its limitations: while exerting strong control over prices, it exerts little control over utilization.

One of the reasons for the long lengths of hospital stay in Japan is the lack of integration between hospitals and primary care practitioners. Effective referral systems between specialized hospitals and primary care practitioners are desperately lacking. Practitioners have been reluctant to refer their patients to secondary or tertiary hospitals and hospitals have been reluctant to discharge the patients to be taken care of by local practitioners. Keeping bed occupancy full has historically been of supreme importance because under the fee-for-service reimbursement any vacant beds mean the deficit in revenue. Such situation is most evident in psychiatric and geriatric hospitals.

There have been attempts to develop American-style ‘open system’ hospitals in Japan with which practitioners continue to serve as the attending doctors of inpatients, but many of them did not take root [2] because there remains a strongly held idea that a single health care institution should ideally provide complete sets of care to a patient. The government has been making efforts to shorten the length of stay (LOS) in hospitals and/or increase the referral rates through economic incentives of the fee schedule (e.g. by increasing the reimbursement for hospitals with shorter LOS and higher referral rates) to help reduce the geographical variance of length of stay particularly among the elderly [3]. However, the economic incentives alone had limited success. Later, the government started a model project of regional information network of strokes in the 1990s. In the model project, hospitals discharging a stroke patient had to co-ordinate the transfer of them to a local public health center [4]. This model project was discontinued by the time the Long-term Care Insurance (LTCI) was introduced in 2000 [5].

### In-hospital versus inter-provider critical paths

The concept of the ‘critical path’ was originally developed in the USA in the 1950s as a process management tool to examine and improve the efficiency of inter-dependencies between various activities undertaken in a specific project [6]. The approach was later adapted to in-hospital management by Karen Zander in 1988 in the wake of the introduction of diagnosis-related group (DRG) reimbursement to acute care hospitals [7]. Japan’s LOS was much longer than the OECD average and the government attempted a variety of measures to shorten it. For example, in 1996, ‘hospital discharge planning’ was introduced to Japan’s national fee schedule. A hospital received additional reimbursement by providing a newly admitted patient with a discharge plan specifying the expected LOS. Although, the discharge plan was not binding, it contributed to shorten the LOS by preparing the patient when he/she would expect to discharge. Hospital discharge planning can be seen as a precursor of an ‘in-hospital’ critical path; government policy aiming to shorten LOS had been strengthened and critical paths were gradually introduced to hospital management mainly to streamline the in-hospital care and shorten the LOS. In 1998, a group of doctors organized to form the ‘Critical Path Study Group’, which later developed into today’s ‘Japan Society of Health Care Management’ [8].

Soon, it became realized that an in-hospital critical path could also be applied to integration among different health care providers and pioneering work started in some areas. However, integration among different providers is difficult in Japan since contractual arrangement between insurers and providers is not authorized by law (unlike, say, the staff-model Health Maintenance Organizations in the USA). Subsequently mutually rivaling providers must cooperate by sharing the patients’ information. In 2005, a Ministry of Health, Labour and Welfare (MHLW) research project was conducted to investigate such pioneering work [9]. The project revealed that in as many as 20 areas inter-provider critical paths were already in place and operational. These findings provided important evidence leading to the inclusion of disease-oriented inter-provider critical paths into Regional Health Planning (RHP) in 2008.

### Development of Japan’s health planning

Japan’s health care system used to be characterized by its *lack* of planning: there was no control over hospital constructions or location of expensive medical equipment. Consequently, Japan has become a country with one of the highest rates of use of medical devices and hospital beds per capita (the number of CT and MRI scans per million population was 97.3 and 43.1; and hospital beds per thousand population was 13.8 in 2008 according to OECD Health Data, by far the highest in OECD countries). Japan’s post-War health policy had been dominated by the Japan Medical Association (JMA) led by a powerful and charismatic leader, Dr. Taro Takemi (1904–1983, JMA presidency: 1957–1982) [10]. Under his leadership the JMA became a powerful pressure group that advocated for professional self-governance of doctors and the exclusion of governmental intervention. It was only after Takemi stepped down in 1982, when the then Ministry of Health and Welfare (MHW) fostered the idea of planning in health policy.

In 1984, the MHW first proposed the introduction of RHP, which was enacted into the Medical Care Act in 1986, mandating each of 47 prefectures to design its Regional Health Plans by 1988. The intent and objectives of the introduction of RHP was mainly to control the growth of hospital beds. Until then, hospital construction was approved as long as applications were compliant with legal requirements. Boosted by the robust economy at that time, hospital construction was booming (Japan’s acute care hospital beds increased by 50% in just ten years between 1978 and 1988) and there was fear of undue inflation of health care expenditure due to the ‘Roemer’s law (a built bed is a filled bed)’ [11]. The new RHP authorized prefectural governments to refuse applications for new hospital constructions where hospital beds were already in over supply. However, little emphasis was placed on how health care should be provided in the RHP.

In April 2008, the Medical Care Act was further amended and the prefectural governments’ initiative over the RHP was strengthened. The new RHP was required to include the ‘disease-oriented’ critical paths to facilitate the “role sharing and effective integration” among different levels of providers and secure a ‘seamless’ provision of care ranging over the primary, secondary and tertiary care as well as home care. For a long time, there has been poor specialization and integration among providers and it was common to see that a stroke patient admitted to a hospital with no recuperative rehabilitation ward was kept in the same hospital after the acute phase without being referred to other appropriate facilities. Referrals among hospitals or doctors tended to be confined among doctors of the same academic clans (informal alumnae networks of the apprenticeship under the same professor) and outsider doctors occasionally found it difficult to choose appropriate referring facilities. There has been a growing understanding that disease-oriented inter-provider critical paths are the best way to ensure that each patient will receive the ‘right’ care at the ‘right’ time, and also to achieve the quality and efficacy of care.

Pursuant to the Medical Care Act, inter-provider critical paths were required to be developed on a disease-oriented manner for at least four disease categories and five health care systems [cancer, acute myocardial infarction (AMI), diabetes, cerebrovascular accidents (CVA), emergency care, rural care, perinatal care and pediatric care]. The new RHP was required to specify which health care providers in the prefecture had certain treatment functions [such as cardiac care unit (CCU) or recuperative rehabilitation] and in what role of the disease-oriented critical paths could the provider be able to provide. For example, in the case of the critical paths for CVA, a medical center with an SCU (stroke care unit) would receive first line treatment; other hospitals would then support a recuperative rehabilitation program by being capable of accepting referrals from the specialist medical center; whilst nurse visitors would then be capable of providing home-based rehabilitation services of the LTCI, and so forth. A special emphasis was placed on the CVA critical path because the CVA is a disease which requires different types of care in acute, recuperative and chronic phases and hence an effective integration is of crucial importance [12].

### Promotion through the fee schedule

The disease-oriented inter-provider critical paths were also promoted through the national mandatory fee schedule. Japan has a universal health insurance system and prices of each service are meticulously set by the national uniform fee schedule [13]. The fee schedule is revised every two years and certain prices are introduced to encourage (or discourage) certain practices. In the 2006 fee schedule, a new fee “regional inter-provider care planning fee (priced at 15,000 yen or $120 according to the rate in 2006: $1=¥124.34)” was introduced. Hospitals can charge this fee for reimbursement upon a discharge of a patient with hip fracture by networking with other local providers to refer the discharged patient (plus conducting an ADL appraisal). In the 2008 fee schedule, the diseases covered by the scheme were expanded to include CVA. To qualify for reimbursement, networking providers must be listed in the critical paths for CVA in each prefectural RHP and networking providers must have regular meetings at least three times a year. The Institute of Health Economics and Policy (IHEP) conducted a questionnaire survey on a total of 625 hospitals fulfilling the requirement for this fee in December 2009 and received responses from 232 hospitals (37.1%) [14]. On average each eligible hospital had a network with 14.5 hospitals (9.1 hospitals with recuperative rehabilitation ward) and 1.3 clinics. More recently, in the 2010 fee schedule, the extent of networking providers was expanded to include LTC facilities and home care agencies.

### Role of public health centers

Although most of the local networks of providers developed voluntarily, local public health centers (PHCs) took initiatives in networking in some areas. PHCs are administrative branches of local governments endowed with various administrative authorities delegated by the Regional Health Act. There were a total of 494 PHCs as of April 2010 and each PHC has its geographical areas of jurisdiction, which overlap with the zoning of the RHP [15]. Therefore, PHCs are expected to act as a coordinator for disease-oriented critical paths of the RHP. Historically, PHCs have been a forefront of primary and secondary prevention but the importance of tertiary prevention (disease management) for chronic diseases is growing. If PHCs are to provide disease management, networking with local providers will be essential. Also, since PHCs have, by law, authority to oversee all health care facilities in the jurisdiction, PHCs are in a better position to coordinate services between the often-rivaling local providers. For example, PHCs may provide a venue for regular meeting for networking providers or act as a liaison with local medical associations.

### Regional health planning guidelines

To help prefectural governments develop RHP, MHLW issued a guideline [16] to help assist prefectural governments with developing disease-oriented RHPs. The guideline emphasizes: “as a policy, RHPs must ensure ‘*seamless*’ health care by *integrating* the specialized functions of health care providers in the region. To achieve this, RHPs must list-up the *names* of providers and their respective specialized functions. Also RHPs should include objective indicators by which quality of care in the region can be evaluated (such as structure, process and outcome indicators [16, p. 28])”.

The disclosure of provider’s names in the RHP was a radical departure from the long-held policy that restricted the advertising of health care facilities. To ensure accuracy, prefectural governments are authorized to conduct surveys on health care providers to grasp the functions of individual providers. To assist the development of RHPs, a national database collecting all health insurance claims nationwide was established by the MHLW.

The RHP guideline places a particular emphasis on CVA because it is a disease “requiring good integration of different types of care such as medical care, long-term care and social care most”. The guideline emphasizes an understanding of the epidemiology of CVA, effective treatment in the acute and rehabilitation phases of care, and the need for evaluating outcomes. The following is the excerpt of the guideline using the part of CVA as an example.

#### Understanding the epidemiology of CVA

Japan had a notoriously high incidence and prevalence of CVA due in large part to high prevalence of hypertension and high salt intake [age-adjusted mortality of cerebrovascular disease of Japan was by far the highest among OECD countries in 1960: 295.2 per 100,000 population or nearly 100 higher than the second (Germany 198.3) according to OECD Health Data]. Not only can CVA be life-threatening, it could lead to disability and subsequent deteriorated quality of life. However, the severity of CVA and its consequences can be minimized through a better coordination of different phases of care. CVA accounts for approximately 11% of emergency patients brought in by ambulances (approximately 330,000 [17] and a total of 1.37 million patients were estimated to be under treatment for the disease in 2005 [18]. CVA is the third cause of deaths accounting for 11.8% (approximately 130,000) of total deaths. It is also important in the social context because in many cases it leaves disability. CVA accounts for approximately 25.7% of beneficiaries of the LTCI and 30% of the bed-bound elderly [19]. Approximately 23% of the new CVA cases ended up being bed-bound in the first month and 19% in the first year according to a local stroke registry [20].

#### Acute phase of treatment of CVA

Prompt transfer to appropriate health care facilities is of crucial importance for the initial acute phase of treatment. However, only 37% of the new cerebral infarction cases were started treatment at the facilities appropriate for CVA within three hours of the onset according to a survey [21]. The study found that improper selection of transferring facilities was part of the reason for the delay in treatment. The initial delay in treatment can be crucial, particularly because the advanced CT is able to detect the very early phase of cerebral infarction, thereby enabling the timely administration of thrombolytics. Ideally, patients should be brought into a facility with SCU (stroke care unit) within two hours and the initial treatment such as administration of thrombolytics within one hour of the arrival.

#### Rehabilitation phase of CVA

For patients who survive the acute phase, early and intensive rehabilitation becomes essential. Such recuperative rehabilitation may initiate in the first 24 hours of the onset because early intervention results in better outcomes [22]. Recovery of lost motion can be expected up to three to six months but once the paralysis is completed, it cannot improve any more. Recuperative rehabilitation is continued as long as improvement is expected but the decision to switch to the chronic phase becomes necessary to avoid unnecessarily prolonged hospitalization. A patient will be discharged when his/her maximum recovery is achieved [23].

After discharge, patients will migrate to the chronic phase of rehabilitation at home or at an LTC facility. In this phase, the LTCI replaces the health insurance and a variety of services are managed by certified care managers. Visiting nursing services and visiting rehabilitation services are staples of the LTCI.

#### Outcome and evaluation

The RHP must incorporate targets to be achieved in the planned period (five years). The targets are expressed in quality indicators measuring the structure, process and outcomes of care—for example:

- Structure: the number of hospitals fulfilling the requirements for t-PA (tissue plasminogen activator) thrombolytic treatment.
- Process: the number and percentage of patients brought to hospitals with primary diagnosis of ischemic stroke who received t-PA administration.
- Outcome: the survival rate and the level of ADL one year after the onset of CVA as examples of outcome indicators.

Prefectural governments are required to analyze their quality indicators to evaluate the effectiveness of the RHP. The first RHP covers the five-year period between 2008 and 2012 with the final evaluation to take place in 2013.

## Description of the policy development in practice

We now turn to examples of how the inter-provider clinical paths have been developed in practice. The examples presented here are drawn from an ongoing MHLW-funded research study led by the authors looking at how standardization of data formats and EHRs (electronic health records) can support disease-oriented critical paths.

### Example 1: RHP for diabetes in Tochigi prefecture

**Figure 1:**
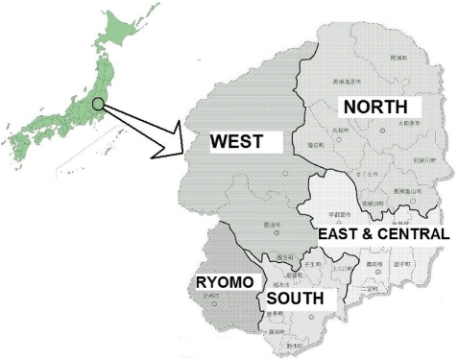
Subregions of Tochigi prefecture RHP.

In April 2008, as with all the 47 prefectures of Japan, an RHP was developed in Tochigi prefecture (population approximately two million) [24]. Tochigi prefecture is a land-locked prefecture 70 miles north of Tokyo. Its RHP divides the prefecture into five regions (Figure 1) and lists up the providers based on their functions (Table 1).

The following table describes the number of providers specialized for diabetes care. The providers are further divided into the variety of phases of diabetes care that they are able to provide. The entire list is published through the prefecture’s website together with the links to individual provider’s website. The list is intended to facilitate inter-provider referrals to ensure optimal treatment to diabetic patients [25].

### Example 2: RHP for diabetes and other diseases in Chiba prefecture

Chiba prefecture occupies the most of Boso Peninsula adjacent to Tokyo and has a population of about six million people. Although it is part of the greater Tokyo metropolitan area, its health care is notoriously underserved (for example, the number of practicing doctors was 161 per 100,000 population, far below the national average of 213 in 2008 or the third lowest among 47 prefectures [26]). The Sanbu-region of Chiba prefecture has had a problem of poor glycemic control among diabetic patients. As a result, the region had a high amputation rate of diabetics, about five times the national average in 1998. Following a new hospital administration under the leadership of a Dr. Hirai, the prefectural general hospital serving as a center of the region—Togane hospital [27]—undertook the development of a model project in the development and use of regional EHR system in 2000 [28]. The project intended to improve the quality of diabetes control by local practitioners through collaboration between local practitioners and specialists of Togane hospital. The EHR system was an indispensable tool for information sharing of patient records between them.

**Figure 2:**
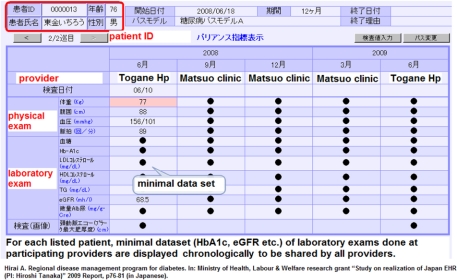
The front page of the electronic health record for inter-provider critical path for diabetes.

The regional EHR system of Togane hospital now manages a dataset of 3200 diabetic patients which is shared with local practitioners. The EHR system developed a minimum dataset with explicit referral criteria related to a number of presenting symptoms [for example, HbA1c levels, eGFR (estimated glomerular filtration rate), urine-albumin, urine-protein, intima-media thickness of carotid artery, and LDL-Cholesterol]. The minimum dataset together with explicit referral criteria were regarded as necessary for critical pathways for diabetes care because these enabled standardized and timely judgment by local practitioners. By sharing the minimum dataset through the EHR, local practitioners have been supported to make more appropriate criteria-driven judgments when referring patients for specialist treatment. Once the patients are stabilized by specialists care by Togane hospital, the critical path approach supports an effective referral back to the referring practitioners. The pioneering work of regional EHR system and the successful adoption of disease-oriented critical pathways attracted attention of the government and prompted it to legislate disease-oriented critical pathways in prefectural RHPs in 2008 (Figure 2).

A key element of the design of the intervention has been to support improvements in the skills of practitioners through peer-review study groups (a process called *staged diabetes management*). As a result, the number of diabetic patients provided with insulin injection under management of practitioners increased dramatically—from eight patients in one clinic in 1998 to 450 patients across 36 clinics by 2007. This has reduced the burden on doctors in Togane hospital where diabetes specialists have been in short supply.

A further seven projects are ongoing in chronic disease management across Chiba prefecture, each building explicit criteria for inclusion, intervention and outcome measurement. For example, one project aimed at tertiary prevention of chronic kidney disease (CKD). Early detection of patients at risk of kidney failure is possible by incorporating a regular kidney function test (such as eGFR, or estimated glomerular filtration) into critical pathways for diabetes and setting an explicit criteria (such as an inclusion criteria of eGFR≤30). The project demonstrated that dietary interventions on phosphate and sodium intake plus antihypertensive medication brought about the reduction of new cases of dialysis.

### Example 3: RHP for CVA and strokes in Aichi prefecture

Aichi prefecture is located in the central part of Japan and is known as a major industrial area particularly for its auto industry with population of approximately 7.4 million. In Aichi prefecture, an EHR system called ‘CVA inter-provider critical paths support system’ was established in 2006 by a non-profit organization ‘Tokai Net Iryo Forum’ with funding from the Ministry of Economics and Industry [29]. The system provides data exchange between acute care hospitals, rehabilitation facilities and long-term care facilities. The project developed a minimum dataset for evaluation of patient conditions to be shared between the providers (such as consciousness level measured in Glasgow Coma Scale) and standardized it based on the uniform terminology called Health Level 7 (Ver2.5 Clinical Document Architecture Release 2) [30].

The critical path begins after a patient with CVA that was admitted to an acute care hospital and has survived the initial phase. The patient is then asked by doctors where he/she wants to have his/her medical record to be included in the EHR system. If the patient agrees to be included in the EHR system, then the acute care hospital sends the patient’s information and data to any of the participating recuperative rehabilitation hospitals. In consultation with the patient, the acute care hospital decides to which rehabilitation hospital they want to transfer the patient. The receiving rehabilitation hospital then conducts an assessment of the level of activities of daily living (ADL) which will be fed back to the acute care hospital to help them monitor the recovery of the patient. Likewise, when a patient is then moved-on to be cared for by the community-based chronic care service, a further assessment of ADL is carried out which is fed back to both the acute care and rehabilitation hospitals that had provided care along the pathway.

In 2007, a total of 25 facilities (10 acute care facilities, nine rehabilitation care facilities and six terminal care facilities) exchanged patient data and by 2008 this number grew to 31 as four acute care and two rehabilitation facilities were added. During one year (November 2007 to October 2008) a total of 292 CVA patients were included in the system, of whom 71 completed through to the chronic care phase.

**Figure 3:**
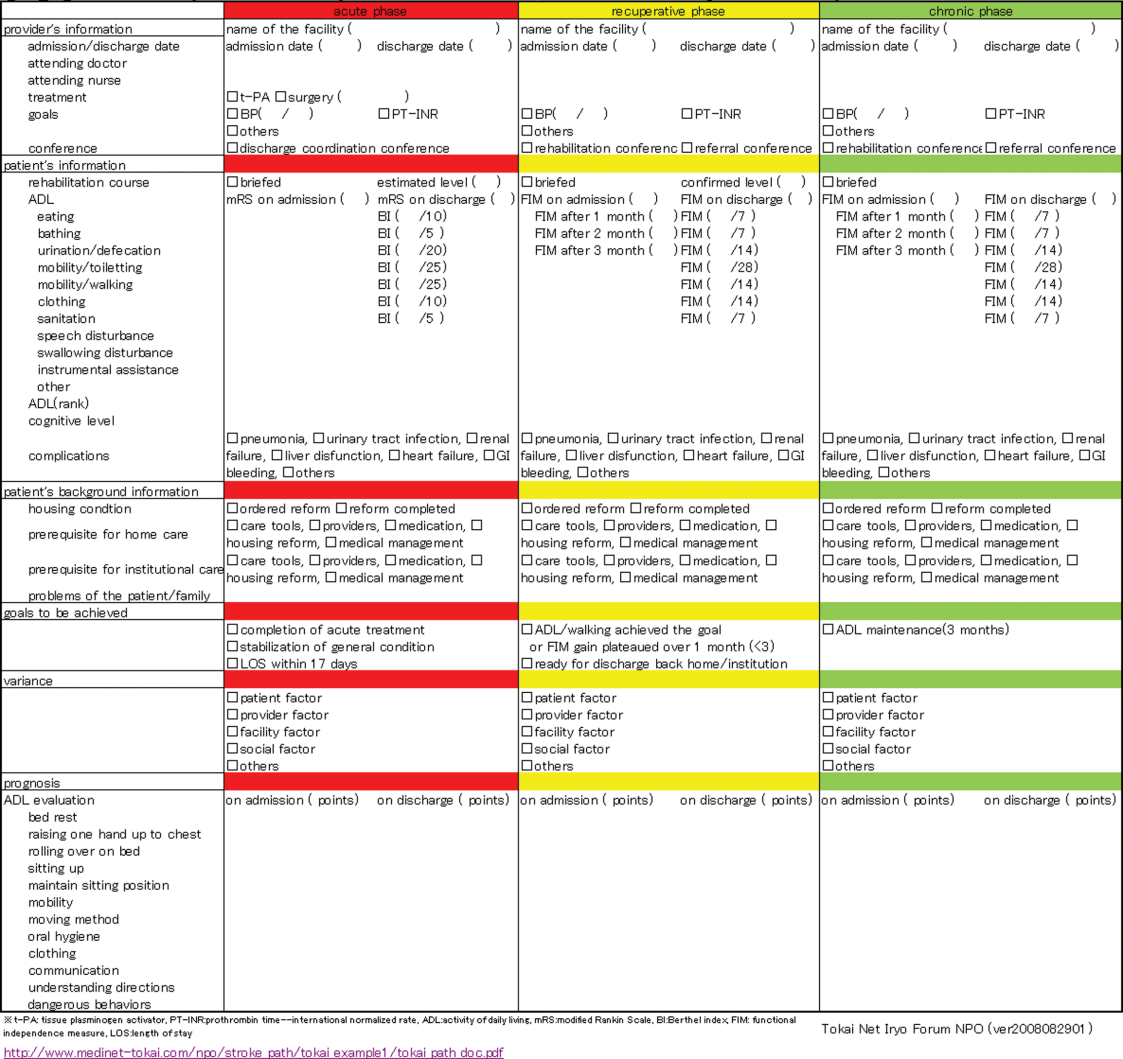
Inter-hospital critical path for strokes (to be shared by different providers).

**Figure 4:**
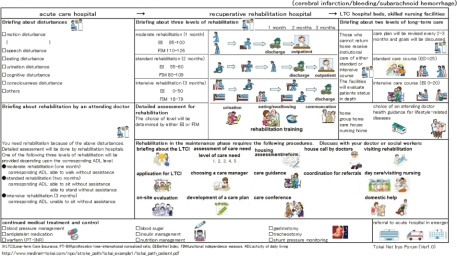
Inter-hospital critical path for strokes (for paMr./Ms.).

Using this system, patients discharged from the acute care hospital receive their follow-up outpatient treatment both from the acute care hospital and via an exercise program provided by a rehabilitation clinic. Both the hospital and the clinic are able to share his/her health records on an ongoing basis. The clinician’s version of the inter-provider critical path to be shared by providers is shown in Figure 3 and the patient‘s version in Figure 4. The professional version is a form of the shared EHR.

### Example 4: Web-based information exchange in Kagawa prefecture

Kagawa prefecture, the smallest of 47 prefectures in its area size facing the inland sea with approximately one million population, has developed a distance health care system called K-MIX (Kagawa Medical Internet eXchange) that originally supported the provision of perinatal care to pregnant women when it was started in 2003 [31]. K-MIX is an application service provider system, which provides a data exchange program online without charge and allows any health care provider to access information via the internet without a particular software at low cost and with high safety. K-MIX facilitates the flow of medical information, such as patient information or medical images, between hospitals and clinics. K-MIX is managed by the Kagawa Medical Association and over 90 hospitals and clinics, including those in adjacent prefectures, have become users.

**Figure 5:**
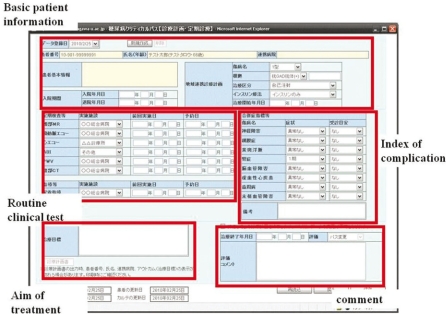
Web page of K-MIX for data input of critical path for diabetes.

K-mix has been expanded to include various critical paths for other than perinatal care such as strokes and diabetes. In 2009, Kagawa University Hospital was able to start the development of an inter-provider critical path for diabetes using the K-MIX system. Hospitals and clinics are able to share patient data (basic patient information, routine clinical test, index of complication, estimation) over the K-MIX web pages and the input data can also be integrated with other K-MIX systems. Figure 5 provides an illustrative example of an input page of a diabetic patient on K-MIX.

### Outcome of inter-provider critical paths

The number of hospitals fulfilling the requirement for reimbursement of ‘regional inter-provider critical path’ has increased steadily starting from 76 in 2006 when it was introduced to 209 in 2007, 405 in 2008 and 613 in 2009 (cf. Japan’s total number of acute hospitals is 7714 as of October 1, 2008) according to the report submitted to the Central Social Insurance Health Care Committee (CSIHCC) which is responsible for the fee schedule revision [32].

Between November 2008 and January 2009, a survey on all hospitals was undertaken to investigate how much inter-provider critical paths were used. The survey was conducted as a questionnaire via regular mail or internet and 546 providers responded (response rate 10.2%) [33]. Of the respondents, 177 of them responded that they were already using the inter-provider critical paths (151 of them relied on paper forms and only 26 of them used electronic data exchange). On a disease-specific basis, 144 used critical paths for CVA followed by 111 for hip fractures (others were cancer: 17, diabetes: 13, AMI: 11). As for the potential for the quality improvement, 65.2% of respondents agreed that the regional integration critical paths will contribute to the improvement of quality of care while only 5.5% denied it. Also, 85.2% of respondents believed that it would be necessary for effective regional integration while 11.9% denied it.

Later CSIHCC conducted an evaluation survey in June 2009, sending a mail questionnaire to randomly selected 2058 hospitals with 744 responses (response rate: 36.1%), of which 138 hospitals were those fulfilling the requirement for the regional inter-provider critical path. Contrary to expectations, it was reported that stroke patients who were part of the inter-provider critical path had a *longer* LOS than those otherwise (33.3 days vs. 30.0 days) [34].

The Institute of Health Economics and Policy (IHEP) also conducted a similar survey on all hospitals fulfilling the requirement for the regional inter-provider critical path (n=625) in December 2009 with 232 responses (response rate: 37.1%) and came up with a similar result: there was a longer LOS in those hospitals applying the integrated critical path than those which did not (32.9 days vs. 27.4 days, standard deviation 9.5 days vs. 10.6 days, respectively).

Overall, there is no definitive evidence as yet that the inter-provider critical path can reduce LOS. However, without baseline information on LOS before intervention, the CSIHCC and IHEP surveys cannot answer this question without more longitudinal data, and that the results have also not been adjusted for the case-mix of the patient. Moreover, there is a study on the inter-provider critical path on hip fracture patients concluding that the apparently shorter LOS of acute hospitals just cost-shifted part of their cost to chronic hospitals [35]. It is premature, then, to draw any definite conclusion on the impact of the adoption of integrated clinical paths in Japan at this stage.

## Discussion and conclusion

The concept of ‘critical path’ was originally developed in the USA as a project management tool and was later adopted to in-hospital management in the wake of the introduction of DRG reimbursement. Recently, the critical path came to be used for inter-provider integration on a regional level, specifically in the management of people with chronic conditions [36]. In other countries, such forms of integrated care have typically involved case management or highly co-ordinated care strategies such as like PRISMA in Canada [37]. Japan’s new RHP does not involve the application of these concepts, nor does it involve a fully-integrated model like the social HMO in the USA [38]. Rather, the context of the Japanese health care system makes the concept of ‘disease-oriented regional inter-provider critical paths’ unique in its approach, particularly since it was adopted as a national policy and all 47 prefectures developed their RHPs simultaneously.

This paper has drawn on emerging information from the authors’ research project on the use of EHR for the purpose of standardizing the data format of the inter-provider critical path. It has reported its latest findings and achievements on how EHR would enhance the inter-professional integration. However, the use of EHR is merely a tool to achieve the purpose and is by no means a prerequisite for effective inter-professional integration. The government’s new initiative for RHP does not require the use of EHR. Ironically enough, in the course of our work, we came to believe that personal networking and mutual trust was far more important than the IT in this regard. The examples we reported in this article were possible not because of IT but because of strong personal networking developed in the long-term. EHR is definitely an effective tool to supplement the human network but can never replace it. This fact is echoed in the requirement for regular meetings with participating providers to qualify for reimbursement for “regional inter-provider care planning”. Unlike the in-hospital critical path, which is used only by the in-house staff, the inter-provider critical path involves staff of different providers. EHR will enhance, but not guarantee, the success of the inter-provider critical path.

## Figures and Tables

**Table 1.** Number of health care providers for diabetes in Tochigi regional health plan

Name of the region	Primary care	Special care	Acute complication	Chronic complication		
				Nephropathy	Retinopathy	Neuropathy
North	33	13	1	7	12	12
West	21	8	0	4	6	9
East-Central	90	15	1	21	16	44
South	58	13	2	12	9	17
Ryomo	28	7	1	7	9	9
Total	230	56	5	51	52	91

## Reviewers

**Jason Cheah**, Dr, MBBS (Singapore), MMed (Public Health), MSc (Wales), MBA, FAM (Singapore), Chief Executive Officer, Agency for Integrated Care, Republic of Singapore

**Han Kwee HO**, Director primary and Community care division, Health Services Group, Ministry of Health HQ, Singapore; Adjunct Assistant Professor, Department of Community, Occupation and Family Medicine, National University Health System, Singapore

**Shinichi Tanihara**, MD, PhD, Associate Professor, Department of preventive medicine and public health, Faculty of medicine, Fukuoka University, Fukuoka 814-0180, Japan
